# Salivary Gland Transplantation as a Promising Approach for Tear Film Restoration in Severe Dry Eye Disease

**DOI:** 10.3390/jcm13020521

**Published:** 2024-01-17

**Authors:** Jiayi Chen, Tianyi Bai, Jiazeng Su, Xin Cong, Lan Lv, Louis Tong, Haozhe Yu, Yun Feng, Guangyan Yu

**Affiliations:** 1 Beijing Key Laboratory of Damaged Ocular Nerve, Department of Ophthalmology, Peking University Third Hospital, Beijing 100191, Chinayuhaozhe@bjmu.edu.cn (H.Y.); 2 National Engineering Laboratory for Digital and Material Technology of Stomatology, Beijing Key Laboratory of Digital Stomatology, Department of Oral and Maxillofacial Surgery, Peking University School and Hospital of Stomatology, Beijing 100081, China; 3 Key Laboratory of Molecular Cardiovascular Sciences, Beijing Key Laboratory of Cardiovascular Receptors Research, Department of Physiology and Pathophysiology, Peking University School of Basic Medical Sciences, Ministry of Education, Beijing 100191, China; congxin@bjmu.edu.cn; 4 Department of Ophthalmology, Beijing Tong Ren Hospital, Capital University of Medical Science, Beijing 100730, China; 5 Cornea and External Eye Disease Service, Singapore National Eye Center, Singapore 168751, Singapore; louis.tong.h.t@snec.com.sg; 6 Ocular Surface Research Group, Singapore Eye Research Institute, Singapore 169856, Singapore; 7 Eye-Academic Clinical Program, Duke-National University of Singapore, Singapore 169857, Singapore; 8 Department of Ophthalmology, Yong Loo Lin School of Medicine, National University of Singapore, Singapore 119228, Singapore

**Keywords:** dry eye disease, transplantation, salivary gland, surgical technique

## Abstract

With increased awareness of dry eye disease (DED), a multitude of therapeutic options have become available. Nevertheless, the treatment of severe DED remains difficult. In a patient whose DED is related to the loss of lacrimal function without severe destruction of the salivary glands, autologous transplantation of the latter as functioning exocrine tissue to rebuild a stable tear film is an attractive idea. All three major and minor salivary glands have been used for such transplantation. Due to the complications associated with and unfavorable prognosis of parotid duct and sublingual gland transplantation, surgeons now prefer to use the submandibular gland (SMG) for such procedures. The transplantation of the SMG not only has a high survival rate, but also improves dry eye symptoms and signs for more than 20 years post-surgery. The regulation of the secretion of the transplanted SMG is critical because the denervated SMG changes its mechanism of secretion. Innovative procedures have been developed to stimulate secretion in order to prevent the obstruction of the Wharton’s duct and to decrease secretion when postoperative “epiphora” occurs. Among the minor salivary glands, the transplantation of the labial salivary glands is the most successful in the long-term. The measurement of the flow rates of minor salivary glands and donor-site selection are critical steps before surgery.

## 1. Introduction

Dry eye disease (DED) is a multifactorial disease of the ocular surface characterized by a loss of homeostasis of the tear film and is accompanied by ocular symptoms, in which tear film instability and hyperosmolarity, ocular surface inflammation and damage, and neurosensory abnormalities play etiological roles [1]. In cases of severe DED with no functional lacrimal glands, the transplantation of salivary glands is a good option for improving symptoms and ocular lubrication, as the transplanted salivary glands are functional and provide lubrication and bioactive factors in the tears constantly [2]. The salivary glands used as lacrimal gland substitutes include the three major salivary glands (parotid, submandibular, and sublingual glands) and the minor salivary glands (MSG) [3].

In 1951, Filatov and Chevaljev introduced the concept of parotid duct transposition and conducted eight percutaneous-approach operations [4]. However, clinical practice has shown that this procedure often results in profound epiphora [5], and the length of the parotid duct is frequently insufficient to reach the conjunctival cul-de-sac [6,7].

Autologous sublingual gland tissue transplantation has been attempted in rabbits and humans, with only Murube reporting on a few cases [8]. Unfortunately, due to the absence of vascular anastomosis, patients with severe aqueous-deficient dry eyes have experience significant graft necrosis [9], and there has been a lack of follow-up reports in recent years.

On the other hand, studies have demonstrated the long-term success of using labial salivary glands, which are a type of minor salivary gland [3]. According to a retrospective cohort study by Su et al., MSG transplantation is a simpler, shorter, and less costly procedure compared to SMG transplantation. Moreover, MSG transplantation has shown promising results for refractory DED cases with relatively less severe impairment of the eyes. However, the effectiveness of MSG transplantation is limited for improving the clinical indicators of DED [10].

Therefore, SMG transplantation holds greater advantages for addressing end-stage refractory DED, which represents the most challenging clinical cases. Given the complications and unfavorable prognosis associated with parotid duct and sublingual gland transplantation, surgeons now prefer to utilize the submandibular gland (SMG) for such procedures.

## 2. Indications

The etiology of DED in SMG transplantation includes Stevens–Johnson syndrome/toxic epidermal necrolysis syndrome, followed by acute conjunctivitis, ocular pemphigoid, Sjőgren’s syndrome, lacrimal gland absence (congenital, traumatic, or pathologic), and chemical burns [8,11,12,13,14,15,16,17,18].

## 3. Contraindications

Sjőgren’s syndrome is a major cause of DED. Sjőgren’s syndrome affects not only the lacrimal glands, but also the salivary glands. The typical symptoms of Sjőgren’s syndrome include hyposalivation and xerostomia [19], which are part of the diagnostic criteria of Sjőgren’s syndrome. As one of the major salivary glands, the bilateral SMGs produce about 65–70% of the total saliva in the resting state. Xerostomia is, thus, a possible donor site complication after SMG transplantation, especially for patients who have had bilateral surgeries [20]. On the other hand, the SMG with hyposalivation cannot provide the required amount of secretion for the lubrication of the ocular surface.

Even though Sjőgren’s syndrome was included in the SMG cohort reported by Murube-del-Castillo et al. [12], the later reports stated that Sjőgren’s syndrome was the contraindication of SMG transplantation [21]. In addition, the patients with obvious symptoms of xerostomia or with a whole-saliva flow rate of <0.3 g/min and severe dysfunction of multiple major salivary glands in scintigraphy were listed in the contraindications [18].

SMG transplantation is generally reserved for cases of severe DED and is regarded as a final recourse when conventional ocular treatments have proven ineffective [2]. One set of indications published includes absolute aqueous tear deficiency (Schirmer test 1 mm), conjunctivalized corneal surface, persistent severe pain, and a history of failed previous ophthalmologic treatments [22]. Recent studies have also reported relaxed indications, including persistent and pronounced symptoms of dry eyes, Schirmer’s test <2 mm, tear break-up time (TBUT) of <5 s, and positive cornea fluorescein staining [18].

## 4. Ophthalmic Assessment

To determine the severity of the disease, detailed ophthalmologic evaluations, including previous ophthalmologic treatment history, the assessment of dry eye symptoms, Schirmer test, TBUT, rose bengal staining, and fluorescence staining, should be performed [15].

The assessment should include the severity of the symptoms of DED and the impairment of the patients’ quality of life (QOL) [23,24]. However, due to the multifactorial etiology and the variability of symptoms, the DED symptoms may not correlate with the examination results [25].

The patients for SMG transplantation should have assessments of objective clinical tests, DED symptoms, and QOL [10]. The QOL of five domains, including “dry eye symptom bother”, “impact on daily activities”, “emotional impact”, “impact on work”, and “satisfaction with treatment”, should be recorded before and after SMG transplantation. In a cohort of 51 DED patients, greater surgical benefits were observed in the patients with lower pre-operative QOL scores [10].

To evaluate dry eye symptoms, a questionnaire should be administered preoperatively, and the ocular surface disease index (OSDI) is the most widely used one in DED clinical trials [26]. To evaluate the signs of DED, examinations of visual acuity, corneal epithelial status, and tear volume should be carried out. Corneal transparency is examined by slit lamp biomicroscopy. The Schirmer test without anesthetic is the recommended diagnostic test for severe aqueous deficiency, but its invasiveness restricts it from being a routine testing method [26].

## 5. Preoperative and Perioperative Assessment

### 5.1. Selection of the Donor SMG and Assessment of the Blood Supply

Because of the significantly lower normal secretion of tears by the lacrimal gland, as compared to the normal secretion of saliva by the salivary gland, directly using a normal submandibular gland for transplantation may lead to epiphora. Therefore, the scientigraphy with 99mTc-pertechnetate and total saliva flow rate measurement should be performed before surgery to guide the selection of the donor gland [1]. Using the relatively hypofunctioning submandibular gland as a donor gland can prevent both epiphora and xerostomia [10,13,15,16,17,18,27,28,29].

The SMG grafting needs to be microvascularized, and the reconstruction of its blood circulation is crucial for donor survival. In the reported cases, the revascularization success rate in SMG transplantation was around 92%, lower than that in free flap reconstruction in the head and neck region (around 95%) [11,12,14,16,17,18,30].

Most of the failed vascularization procedures were caused by venous thrombosis [16,17,18,30], and two underlying causes for this have been reported [31]. The anterior facial vein (AFV) and the superficial temporal vein (STV) were the most-often used donor vein and recipient vein, respectively [11,12,13,14,16,17,18,28,30,32]. The mean diameters of the AFV and the SV for 42 cases during SMG transplantation were 2.99 ± 1.33 mm and 1.71 ± 0.59 mm, respectively [31]. This size discrepancy of a bigger donor vein and a smaller recipient vein is predisposed to anastomotic failure [33]. In a small number of cases (9.5% in a clinical study by Su et al. [31] and 6.7% in a cadaveric study by Li et al. [34]), the AFV does not drain the SMG and, thus, using it for venous anastomosis would lead to failure.

To avoid such failure, computed tomographic venography is performed to study the AFV and STV before surgery [31]. On volume-rendered three-dimensional images, the SMG, AFV, STV, and their venous branches (as small as the level-4 branches) can be observed. The venography has shown good sensitivity and specificity in detecting the absence of AFV leading from the SMG and the size discrepancy of the AFV and STV. This would be valuable for donor gland selection between the bilateral SMGs [31].

### 5.2. Perioperative Evaluation

The examination of dry eye symptoms and signs should be repeated after surgery. Postoperative evaluation was suggested to be carried out one and six months after surgery by Sant’ Anna [35] and one week and one, three, and six months after surgery by Luo [36].

Recently, a method to assess transplants over a longer period has been developed [3]. To increase the repeatability of the Schirmer test, the evaluation conditions should keep a comfortable and stable room temperature to avoid the bias caused by physical activity and glandular stimulation [37].

## 6. Considerations in Submandibular Gland (SMG) Transplantation

### 6.1. Traditional Surgical Techniques

The operative team should be composed of a maxillofacial surgeon, an ophthalmologist, and a microsurgery specialist. A typical surgical procedure could be divided into three steps [2,3,8,16,17,18,22].

#### 6.1.1. Step One: Recipient Site Preparation

**Figure 1:**
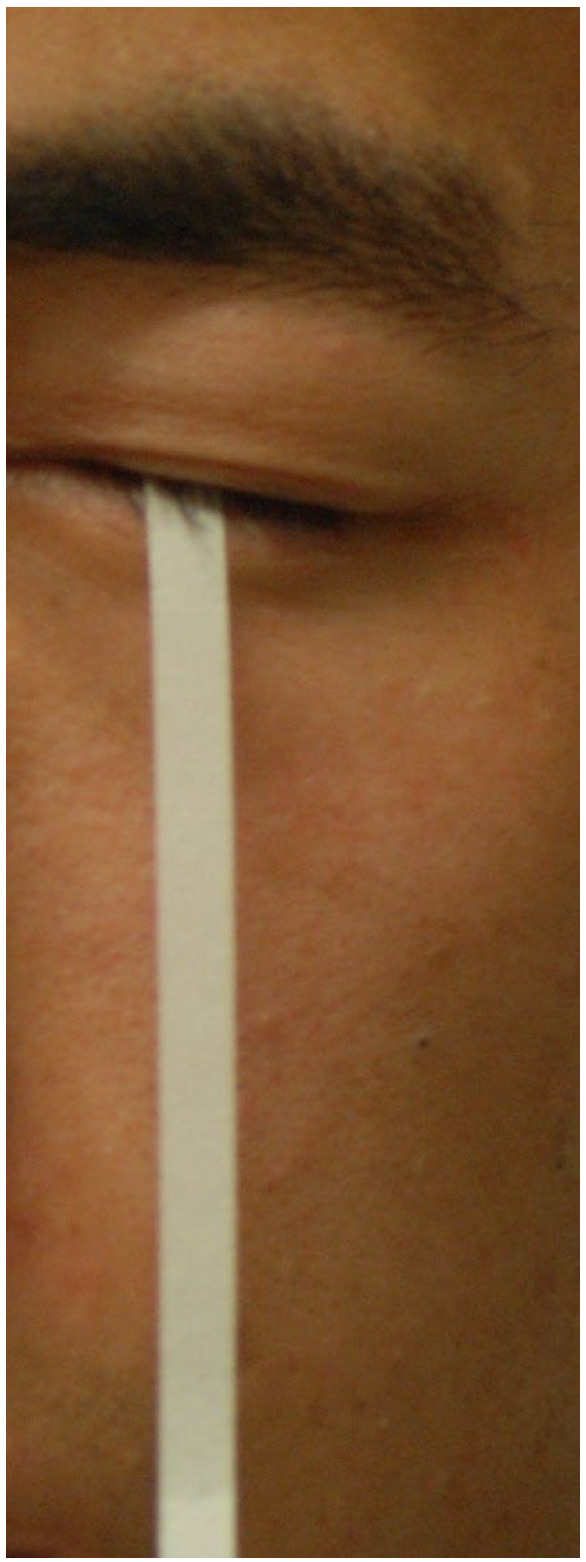
Epiphora after submandibular gland transplantation.

A curved incision from the hairline, along the superior temporal line, and down posteriorly to the pre-auricular region is performed (Figure 1). An enlarged, caudally based temporal flap is lifted from the layer superficial to the superficial temporal vessels, and the vessels in the recipient bed should be preserved intact. Caution is needed during the flap elevation, because the vessels may be very superficial. Some authors have proposed using a doppler probe to identify and mark the vessels before surgery. A simpler and effective method is to lift the flap from the distal to the proximal end. After the superficial temporal vessels are identified in the distal end, the surgeon can safely dissect them to the proximal end, to be used later in the anastomosis [11,16].

#### 6.1.2. Step Two: Donor Harvesting

The donor includes the SMG, the Wharton’s duct, and the related vessels. Conventional surgical access via the submandibular triangle is used and extracapsular dissection is performed to preserve the glandular structures. As in conventional SMG resection surgery for SMG tumors, the distal end of the facial artery and the AFV are severed, preserving the marginal mandibular branch of the facial nerve. The chorda tympani supplying the submandibular gland is then severed, preserving the lingual nerve. Unlike in conventional SMG resection surgery, the proximal end of the vessels, including the facial artery, its accompanying veins, and the AFV needed in anastomosis, should be preserved. Dissection to free up lengths of these vessels should be performed to facilitate the subsequent anastomosis. In addition, the full length of the Wharton’s duct should be dissected intact, and the major part of the duct can be freed by retracting the mylohyoid muscle via cervical access. An incision in the floor of mouth is performed in order to harvest the remaining part of the duct and its orifice. A 2- to 3-mm cuff of the mucosa around the Wharton’s duct orifice should be preserved and included as part of the donor tissue to facilitate the implantation of the duct into the conjunctival fornix. In some cases, the hilar vein of the SMG, if observed during the dissection of the Wharton’s duct, should also be preserved for use during anastomosis [15,30,31].

Before the donor is completely freed, the proximal ends of the facial artery, its accompanying veins, and the AFV are the only connections with the body. It is essential to sever the AFV first. Bleeding from the AFV helps to confirm its availability for later anastomosis. Otherwise, the accompanying veins of the facial artery, or the hilar vein of the SMG, are backups if the AFV does not drain the SMG [15].

#### 6.1.3. Step Three: Donor Transfer

Before transferring the donor, a “pocket” could be prepared in the recipient bed by removing some of the temporalis muscle to create space for the donor [11,13,16]. However, others have felt that the donor could be transferred successfully without removing any recipient bed tissue to decrease the injury of temporalis muscle and to allow the temporal region to remain flat in long-term follow-up [15,30].

The facial artery is then anastomosed with the superficial temporal artery [11,13,14,15,16,30]. Anastomosis of the veins is more complicated. The anterior facial vein, the accompanying veins of the facial artery, and the hilar vein of the SMG have been used by various surgeons [15,30,31]. The AFV is bigger and longer, with a thicker vessel wall than the other two veins, and would make the anastomosis easier and more reliable [31]. In most of the documented cases, the superficial temporal vein was the recipient vein [11,15,16,18,30]. When a reliable superficial temporal vein cannot be found, surgeons bridge the AFV and the external jugular vein using the cephalic vein [15,30].

Subsequently, a small incision is made in the upper lateral conjunctival fornix. Via a subcutaneous tunnel, the distal end of the Wharton’s duct is passed to the conjunctiva, and the cuff of the mucosa around the Wharton’s duct orifice is sutured to form an opening into the conjunctiva fornix. A nylon tube is inserted and left in the Wharton’s duct for at least 7 days after the operation [18].

### 6.2. Techniques of Partial SMG Transplantation

The microanatomy of the human SMG reveals a parallel arrangement of arteries, veins, and ducts in different levels, and their structures are similar within each level of the lobules [38]. Such treelike structure provides the possibility for local transplantation at an anatomical level, which was initially demonstrated in rabbits [39]. For partial SMG transplantation, an additional volume reduction step is performed before or after vascular anastomosis, which effectively addresses the most common complication, epiphora, in traditional total SMG transplantation [10,13,15,16,17,18,27,28,40]. As Figure 1 depicts, the lobules far from the Wharton’s duct and the main stem of the vessels were removed and the reduction volume accounted for about 1/3 to 1/2 of the whole gland in the study. This new technique has since been performed in DED patients with ample SMGs and normal salivatory function, in whom severe epiphora is expected after total SMG transplantation. In a randomized controlled trial with 42 cases, partial SMG transplantation significantly reduced the rate of postoperative epiphora (6/20 vs. 19/22), fewer patients with partial SMG transplantation than total SMG transplantation underwent a subsequent reduction surgery (6/20 vs. 18/22), and no differences in the graft survival rate or other complications compared to traditional surgery were found [41].

### 6.3. Salivary Flow after SMG Transplantation

The secretory function of the transplanted gland, assessed using the lubrication flow rate of the eye, showed a consistent change post-surgery [11,13,14,16,18,30]. Yu et al. [15,18,27] quantified this functional change, dividing it into the following four stages: the transient hypofunction period (the first 2 postoperative days), the temporary epiphora period (3–6 postoperative days), the latent period (1 week to 3 months), and the functional recovery/stable period (more than 3 months). In a cohort of 163 cases with successful SMG and more than 1 year follow-up, the secretion was extremely limited during the initial 1–2 days after surgery. Subsequently, hypersecretion occurred during the temporary epiphora period, when the mean Schirmer’s test value raised to 35 mm/5 min (interquartile range of 23–47 mm). The secretion then declined over the next 3 months during the latent period (mean Schirmer’s test: 1.25 mm/5 min; interquartile range: 0–3 mm). Finally, the secretion flow rate increased again after 3 months (mean Schirmer’s test: 18 mm/5 min; interquartile range: 8.5–30 mm). One year after surgery, the Schirmer’s test value remained stable (mean Schirmer’s test: 19.5 mm; interquartile range: 10–28 mm) [18].

**Figure 2:**
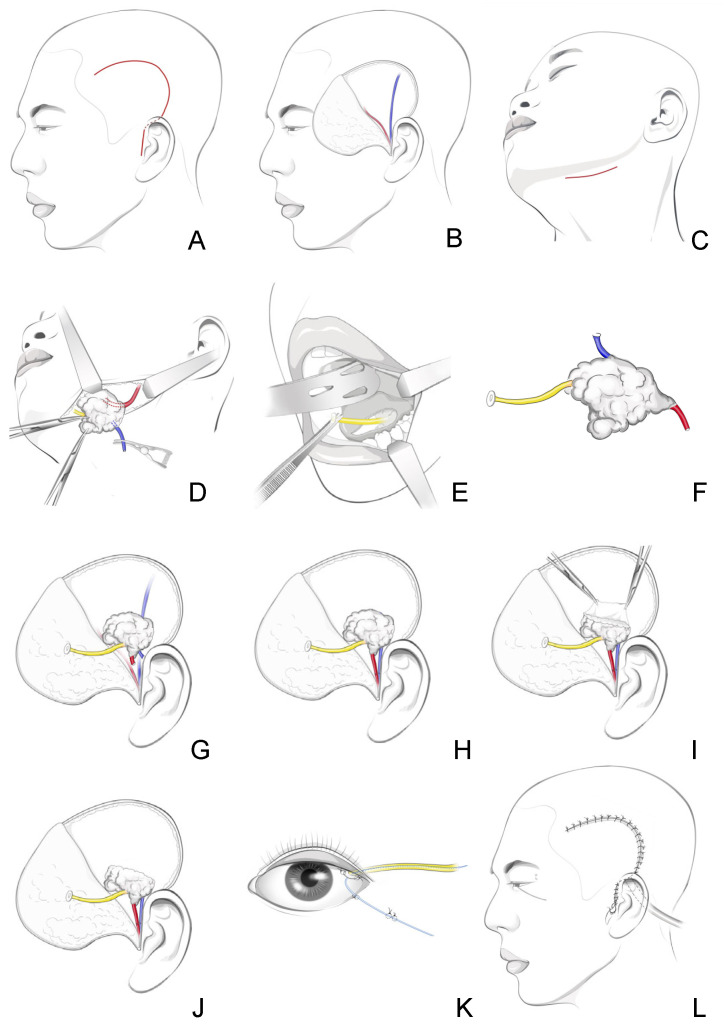
Partial SMG transplantation. (**A**) Incision in the temporal region. (**B**) Preparation of the recipient bed. (**C**) Incision in the submandibular region. (**D**) Harvest of the submandibular gland. (**E**) Harvest of the Wharton’s duct. (**F**) The graft. (**G**) Transplantation of the graft to the recipient bed. (**H**) Part of the gland was removed, with the capsule preserved. (**I**) The graft after volume reduction. (**J**) Vascular anastomosis. (**K**) A nylon tube was inserted and left in the Wharton’s duct. (**L**) Closure of the incision.

The latent period lasted for about 3 months, with little or no lubrication in the eyes. The Wharton’s duct of the transplanted SMG may be obstructed without saliva flow over a long period, leading to surgical failure [18,42]. Beyond the “stable period”, a large percentage of patients complained of too much secretion from the graft, aggravated by warmth or physical activity [28,37]. The rate of severe epiphora (Figure 2), defined as unacceptable lubrication, was 17%, 24.14%, and 60.1% in three long-term follow-up studies [17,18,30]. Severe epiphora resulted in social awkwardness and might lead to microcystic edema of the cornea [17] and even reduced visual acuity [18] and quality of life [10].

**Figure 3:**
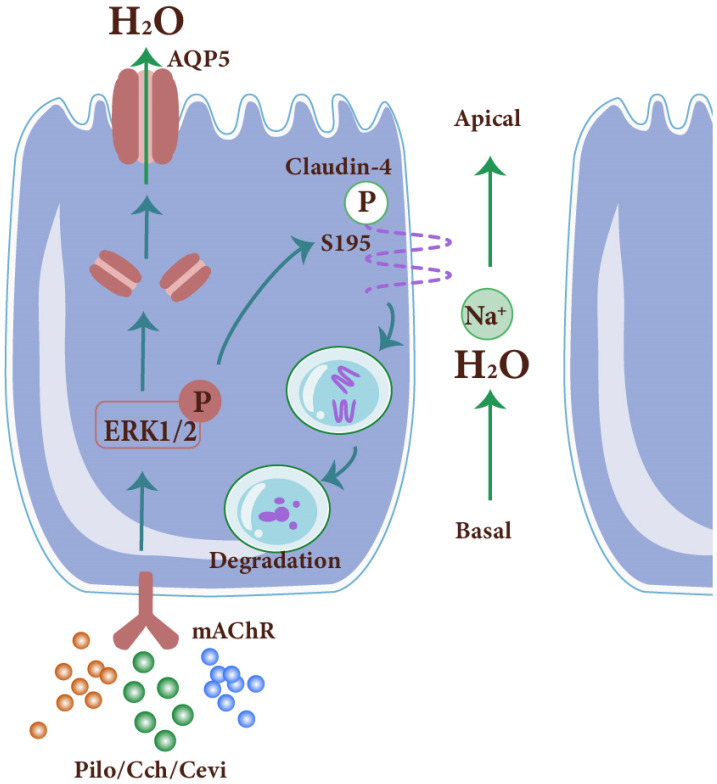
The intracellular mechanism underlying muscarinic acetylcholine receptor (mAChR) in submandibular gland acinar cells. The activation of mAChR by agonists promotes saliva secretion through the aquaporin 5 (AQP5)-mediated transcellular pathway and claudin-4-mediated paracellular pathway, both of which are accomplished in an extracellular-signal-regulated protein kinase 1/2 (ERK1/2)-dependent manner. Pilo, pilocarpine. Cch, carbachol. Cevi, cevimeline. Solid line, transcellular pathway. Dotted line, paracellular pathway.

The secretion of SMGs is primarily controlled by both sympathetic and parasympathetic nerves, with the related receptors, muscarinic acetylcholine receptors (mAChRs) and adrenoreceptors, respectively. Although the nerves of the transplanted glands are not anastomosed, these receptors can still sense the stimulators and trigger the secretory action. By using a rabbit SMG autotransplantation model, Yu’s group found that M1 and M3 mAChRs are decreased in latent period, whereas the carbachol treatment significantly promoted the secretion and M1 and M3 expressions in the transplanted glands [43]. In the long-term transplanted rabbit and human epiphora SMGs, the expressions of the mAChRs are recovered to similar levels as the controls, and the receptors are much more hypersensitive [44,45]. The blockage of the mAChRs by atropine or botulinum toxin A significantly ameliorates the hypersecretion [44,46]. Mechanistically, the activation of the mAChRs directly regulates both transcellular and paracellular pathways through promoting aquaporin 5 (AQP5) expression and relocalization to the apical membranes, as well as tight junction opening, respectively (Figure 3). Thus, modulation of mAChRs might be a promising important strategy to ameliorate the SMG dysfunction that occurs after transplantation [43,47,48].

**Figure 4:**
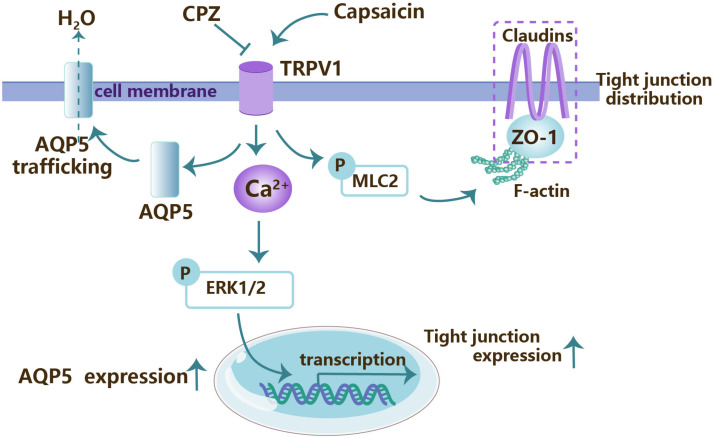
The intracellular mechanism underlying transient receptor potential vanilloid subtype 1 (TRPV1) in submandibular gland acinar cells. The activation of TRPV1 by capsaicin increases saliva secretion by promoting aquaporin 5 (AQP5) trafficking and expression. In addition, TRPV1 activation also regulates the distribution of tight junction proteins in a myosin light chain2 (MLC2)-dependent pathway, as well as their expressions in an extracellular-signal-regulated protein kinase 1/2 (ERK1/2)-dependent manner, thereby increasing the paracellular permeability. CPZ, capsazepine. ZO-1, zonula occludens-1.

Interestingly, the expression and function of transient receptor potential vanilloid subtype 1 (TRPV1) in SMGs was first identified by Yu’s group [49]. The activation of TRPV1 upregulated AQP5 expression leads to AQP5 redistribution in a Ca^2+^-extracellular-signal-regulated protein kinase 1/2 (ERK1/2)-dependent pathway [50]. In addition, TRPV1 activation also modulates the expression and distribution of tight junction proteins in ERK1/2- and MLC2-dependent pathways, respectively, and herein increases the paracellular permeability across the epithelial cells (Figure 4) [51]. Furthermore, the expression of TRPV1 is downregulated in the rabbit transplanted SMGs in a short-term model, whereas the topical application of the TRPV1 agonist capsaicin gel shows a considerable therapeutic effect to promote the secretion and help the glands to overcome the latent period [50,52].

### 6.4. Modulation of Salivary Flow

The hypofunction or hyperfunction of the transplanted SMG would severely impair the treatment outcome. To achieve optimal results, modulating the salivary tear flow is as important as the integrity and vitality of the graft [18].

#### 6.4.1. Administration of Capsaicin and Carbachol

In animal experiments, TRPV1, muscarinic receptors, and their downstream signaling molecules have been observed to be downregulated in the latent period after SMG transplantation. This might partly explain the hypofunction of the graft in this period [50]. In rabbit models, capsaicin (TRPV1 agonist) and carbachol (muscarinic receptor agonist) treatment increased salivary secretion and reduced functional and structural injuries in the transplanted SMGs soon after the operation [50]. These findings have been clinically translated to human patients. Capsaicin cream was applied to the skin covering the graft, and carbachol was subcutaneously injected in the abdominal wall. The secretion of the graft significantly increased after both of these drugs and the effects lasted for more than half an hour. The maximum Schirmer’s test value was 4 mm/5 min and 1050 mm/5 min after capsaicin and carbachol administration, respectively [53].

**Figure 5:**
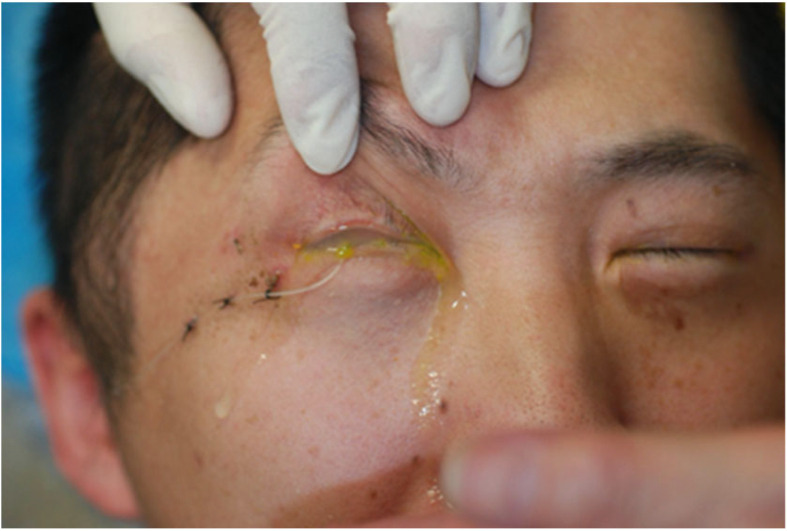
The “internal irrigation” effect of carbachol injection. A very high flow rate of saliva was excreted by the transplanted SMG after carbachol administration, and the viscous depositions were washed away out of the duct along with this strong secretion.

The topical application of capsaicin cream is simple and could be used daily by patients to maintain a basic secretion flow in the latent period. However, the effect of capsaicin was too weak, and salivary stasis and mucus embolus might still form. To supplement capsaicin, carbachol could be added intermittently. The high salivary flow rate after carbachol could “internally irrigate” and flush viscous deposits out of the duct (Figure 5). This combined strategy significantly reduced the rates of duct obstruction (from 18% to 6%) in a cohort of 172 cases with successful SMG transplantation [54].

#### 6.4.2. Techniques to Manage Epiphora

The clinical routines for the management of postoperative epiphora have been formed and practiced. A series of techniques to prevent epiphora, including the use of partial SMG transplantation, conventional reduction surgery, botulinum toxin A (BTXA) injection, or topical application of atropine gel, might be selected, according to the severity of the epiphora [55].

As mentioned above, partial SMG transplantation could be used for patients with ample SMG and normal function, for whom severe epiphora would be expected after a total SMG graft. If epiphora still occurs after partial SMG surgery, or the symptom of epiphora is serious, conventional reduction surgery [13] is recommended. Part of the graft is removed to reduce the size of the graft, which decreases the secretory flow rate. The reduction surgery might be carried out more than once to achieve the desired lubrication [18].

Two other non-surgical methods have been applied to manage postoperative epiphora. The overexpression of M1- and M3-muscarinic acetylcholine receptors (mAChRs) occurred in the resected transplanted SMG in severe epiphora [45]. These hypersensitive mAChRs might be involved in this epiphora. In a clinical trial, topical atropine gel significantly reduced the secretory flow rate of the transplanted SMG, and the effect lasted for around 5 h [55]. The secretion of the transplanted SMG increased with warmth or physical activity [28,37]. The patients with epiphora only during strenuous physical activity outdoors are indications of topical application of atropine gel [55].

The other non-surgical method was local administration of botulinum toxin type A (BTXA), a neurotoxin that inhibits presynaptic acetylcholine release from nerve endings, thereby interfering with nerve impulses [46]. A 25-U BTXA injection could significantly decrease the secretion of the transplanted SMG 1 month after injection, and the effect lasted for at least 3 months. This modality is especially suitable for patients with seasonal epiphora, and BTXA is administrated at the beginning of summer [46,55].

## 7. Results of Clinical Trials

**Figure 6:**
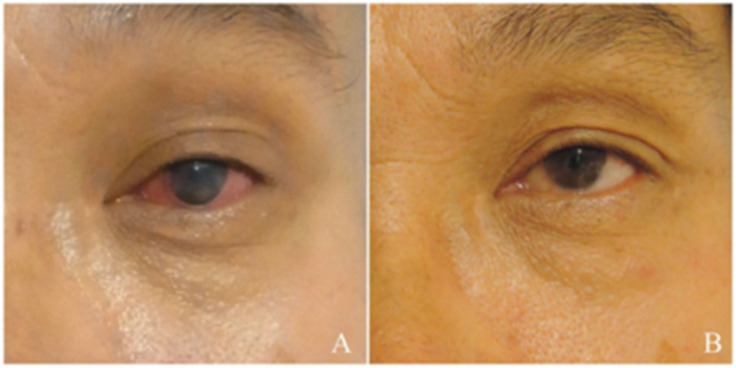
The manifestation of the ocular surface before and after operation. (**A**) Obvious dryness with conjunctival hyperemia before operation. (**B**) Moist ocular surface without conjunctival hyperemia 5 years after operation, demonstrating good long-term results of this procedure.

Zhang et al. reported a cohort with a large sample size and long-term follow-up results in 2019 [18]. A total of 200 severe KCS (keratoconjunctivitis sicca) eyes of 185 patients were treated with SMG transplantation since 1999, with 15 bilateral transplantations. A viable graft with good secretory function was confirmed in 180 eyes, with a success rate of 90% (Figure 6). A total of 163 (88.1%) eyes were followed up for >1 year, and 51 eyes were followed up for >5 year after surgery in this cohort. The most significant changes were reflected in the Schirmer’s test values, which increased from a preoperative level of a median of 0 mm (interquartile range 0–1 mm) to a median of 18 mm (interquartile range 8.5–30 mm) 3 months after surgery and remained virtually unchanged at a median of 19.5 mm (interquartile range 10–28 mm) 1 year after surgery and 18.5 mm (interquartile range 10–28.5 mm) 5 years after surgery. The lubrication secreted by the SMG improved the tear film stability and ocular surface, as shown in TBUT, FL, and BCVA examinations. The mean FL scores reduced from 11.25 ± 1.42 before surgery to 7.25 ± 3.37 (*n* = 151) 1 year after surgery and 5.76 ± 3.67 5 years after surgery. The mean TBUT scores improved from 0 (interquartile range 0–1) before surgery to 3 at both 1 (interquartile range 0–5) and 5 (interquartile range 0–4) years after surgery. The BCVA improved in 85 (56.3%) eyes, with the elimination of blindness 31 patients, remained unchanged in 49 eyes, and decreased in 17 eyes. The relief of symptoms and subjective satisfaction were achieved in 92.6% and 87.7% of the patients, respectively [18].

Geerling et al. reported 42 SMG transplantations in 34 patients, in which 32 grafts (72%) remained viable and functional by the end of the 7-year follow-up [29]. They also launched a cohort study that lasted for 5 years [17]. Among the 15 most severe dry eyes with a successful SMG transplantation, the Schirmer’s test, fluorescein break-up time, and discomfort levels improved compared with 10 dry eyes without surgical treatment. The frequency of using artificial tear substitutes reduced significantly, and five patients were able to eventually stop the application. The reported severity of symptoms was reduced from very severe preoperatively to moderate by the end of the 5-year follow-up. However, visual acuity was not significantly different up to the last follow-up between the two groups.

In addition to the objective examinations, subjective indexes are equally important for the evaluation of the DED treatment effect. Using the dry-eye-related quality of life questionnaire, Su et al. prospectively measured patients’ quality of life both before and 1 year after SMG transplantation in 51 consecutive DED patients from the above cohort. The data confirmed that all five quality of life domains, including dry eye symptom bother, impact on daily activities, emotional impact, impact on work, and satisfaction with treatment, showed significant improvement after surgery. Those patients suffering from more severe DED reaped more benefits from SMG transplantation [10].

## 8. Surgical Complications

### 8.1. Early Postoperative Complications

The most common and severe surgical complication was vascular thrombosis after vascular anastomosis, leading to the loss of graft viability. The rates of this complication were 14.3%, 13.6%, 4.9%, and 7.5% in four independent reports, respectively [16,17,22,30]. Venous thrombosis was much more common than arterial thrombosis [18,30].

The use of preoperative computed tomographic venography to assess and select a donor with a reliable AFV can reduce the rate of venous thrombosis [31]. During the operation, the reliability of the AFV as the donor vein was examined. Before the proximal parts of the facial artery were cut off and the SMG was totally freed, bleeding from the AFV was the indicator that it had collected the venous drainage from the gland. After the gland and the vessels were totally released, heparinized saline was irrigated into the facial artery. Seepage of this solution from the AFV also confirmed that it was reliable as the donor vein [31].

Similar to other microvascular surgeries, the vascular complication mostly emerges 72 h after surgery, especially within the first 24 h [30]. The 99mTc-pertechnetate scintigraphy is recommended as a routine examination around 1 week after the operation to confirm the vitality of the graft [13,15,16,18,30,56]. An uptake of 99mTc-pertechnetate forming a “hot spot” in the temporal region shows a viable graft and primary surgical success. In addition, secretion from the graft during the early postoperative stage would also indicate a viable graft [11,13,14,16,18,30].

In the event of failed microvascular surgery and non-vital grafts, the tissues could be removed or left at the recipient site, avoiding the need for secondary surgery [15,18].

### 8.2. Late Complications

Impaired secretory patterns of transplanted SMG were not rare after the operation. They were called “Wharton’s duct obstruction”, “stenosis of the excretory duct”, “obstruction of the excretory duct”, “infection of the excretory duct”, or “malfunction due to ascending infection” [11,15]. Based on a clinical cohort and animal experiments, Su et al. systematically described the impaired secretory patterns observed after SMG transplantation and coined the term “chronic obstructive sialadenitis of transplanted SMG” to characterize these patterns [42]. Typical clinical manifestations were continuous viscous secretions with decreased flow rates and intermittent gland swelling. The Wharton’s duct showed irregular dilation on sialography. Morphological examinations in the rabbit SMG transplantation models demonstrated that the grafts with impaired secretions had inflammatory cell infiltration surrounding the duct and mucous retention in the ductal lumens. In the clinical cohort, the incidence of this complication was 9.3% (16/172) in the early phase (0–3 months) and 1.3% (2/154) for >1 year after the transplantation. Continuous marked idiopathic decline of the secretory flow rate of the transplanted SMG during the latent period (early phase) or winter season (>1 year) was regarded as the pathogenesis of this complication. The delayed management of obstructive sialadenitis would lead to the severe stricture of the Wharton’s duct and finally to the failure of the surgery. Promotions of the secretion of the transplanted glands during the latent period or winter are recommended to prevent this complication [42].

As mentioned above, epiphora is one of the late complications that influences the quality of patients’ lives. The prevention and management of epiphora is critical for the improvement of the success rate of transplanted SMG for severe dry eyes.

### 8.3. Other Complications

Ranula, sialolithiasis, and duct fistulas were rare after SMG transplantation and were surgically removed [15,30,57]. Xerostomia was reported by 2 of the 53 patients in the study by Wang et al. [30]. Ophthalmologic complications included pseudopterygium, corneal ulceration, microbial keratitis, and salivary lubrication, which resulted in burning sensation, and the incidences ranged from 2.3% to 4.5% [17].

### 8.4. Keratoplasty after SMG Surgery and Its Prognosis

As the primary method used to recover visual acuity, keratoplasty is not indicated for severe DED patients with low vision. However, SMG transplantation improves the ocular surface humidity and offers an opportunity for keratoplasty.

**Figure 7:**
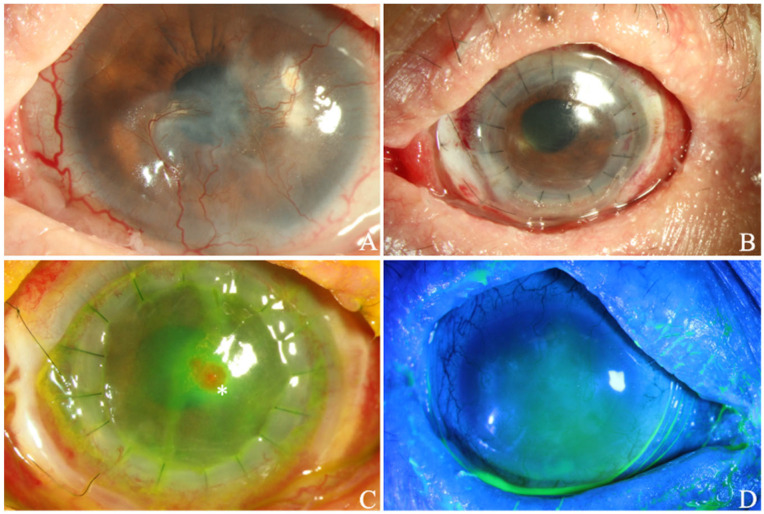
Clinical photographs from a 58-year-old patient with Stevens–Johnson syndrome who underwent submandibular gland transplantation. (**A**) Anterior segment photograph before corneal transplantation. (**B**) Photograph 4 days after transplantation. (**C**) Infection is seen 30 days postoperatively, with small microflora (asterisk) and corneal epithelial ulceration. (**D**) Corneal fluorescein staining of the non-operative eye.

In the cohort study conducted by Zhang et al., lamellar keratoplasty was performed in 15 DED patients who underwent SMG transplantation. After a follow-up of more than 3 years, the grafts remained transparent in five cases. These patients experienced significant improvement in visual acuity and reported an enhanced quality of life [18]. In our group’s experience, a patient with Stevens–Johnson syndrome, whose tear meniscus height (TMH) was 0.1 mm OD/0.15 mm OS, developed an infection 30 days after undergoing penetrating keratoplasty, which may also be caused by epithelial cell defects, as mentioned before (Figure 7). However, the success rate was limited, and further investigation is warranted in order to improve the success rate of keratoplasty.

## 9. Future Directions

### 9.1. Improving the Composition of Secretion of the Transplanted SMG

After long-term studies, the success rate of SMG transplantation was improved, and the secretion of the transplanted SMG could be regulated. However, the secretion compositions of the transplanted SMG differ from natural tears. Moreover, an interesting phenomenon was noticed, showing that the compositions of the secretion from the successfully transplanted SMGs became more similar to that of natural tears, as compared with natural SMG saliva. Potassium and sodium concentrations were increased and, consequently, osmolality increased as well after SMG transplantation [18]. However, the osmolality was still lower than that of natural tears due to the lower level of sodium. The ocular surface is susceptible to minor changes in tear osmolality. Geerling et al. found that hypotonic tears might induce osmotic cell oedema and result in focal epithelial oedema [54,58]. This would be even worse for patients with severe epiphora [17]. The differences in the compositions and osmolality between the secretion of transplanted SMG and natural tears were crucial for patients’ subjective dissatisfaction and decreased visual acuity and might account for the low successful rate of keratoplasty [18]. Future studies should focus on improving the secretion composition of the transplanted SMG to mimic physiological tears. An artificial hypertonic supplement might be a choice to modulate the composition of the transplanted SMG.

### 9.2. SMG Allotransplantation for Patients with Salivary Gland Hypofunction

Ge et al. have shown the technique to be feasible with the administration of an immunosuppressant in miniature swine, and the secretion of the gland lasted for 100 days [59]. The results of experimental studies in rabbits by Almansoori et al. showed that a higher survival probability after allotransplanted SMGs could be expected with the help of mesenchymal stem cell therapy and FK506, without increasing the side-effects [60,61]. However, two GVHD patients undergoing total donor chimerism assessment who received allogenic SMG transplantation still showed signs of organ rejection [62]. In patients with immunological disorders, such as GVHD, Sjögren syndrome, and irradiation-induced lacrimal damage, xerophthalmia is often accompanied by SMG hypofunction [60]. In addition, the function of SMG is seriously involved in some patients with Stevens–Johnson syndrome. In these situations, the SMGs cannot be used as the donor. Efforts are currently underway to investigate whether allotransplantation of SMGs could be an alternative modality.

### 9.3. Reconstruction of the Lacrimal Gland by Tissue Engineering

A tissue-engineered lacrimal gland may be potentially used as an exocrine tissue for patients whose lacrimal gland is entirely nonfunctional. An attempt has been made to reconstruct the lacrimal gland by the induction of whole organs using special cell populations and a ‘scaffold’ for cells to grow on [63,64,65,66,67,68,69]. Cell populations, including lacrimal gland stem cells [70] and epithelial cells [69], have demonstrated the possibility of constructing a lacrimal gland functional unit in vitro. Primary palpebral and fornical conjunctival epithelial cells also show the ability to regenerate to become lacrimal duct epithelium cells [71]. Decellularized tissue seems to serve as a promising scaffold [63,72]. For patients whose lacrimal glands are partially damaged, the regeneration of tissue in situ using drugs, cell therapies, and gene therapies may be preferable, which are less invasive [63]. To address these approaches, clinical trials and in vitro studies are underway.

## 10. Conclusions

In conclusion, multicentric long-term studies with large case examples demonstrate that autologous SMG transplantation is an effective treatment with stable results in severe or refractory dry eyes. The technique can relieve the symptoms of dry eyes and improve the structures of the ocular surface and the quality of patients’ lives. Pre- and intraoperative assessments of the blood vessel status are helpful to avoid vascular thrombosis and improve the survival rate of the grafts. Obstructive sialadenitis of the transplanted SMG and epiphora are the main postoperative complications. The regulation of the secretion of the transplanted SMG by the agonists and antagonists of the salivatory-secretion-related receptors, including mAChRs and TRPV1, can effectively decrease the incidences of these complications. The improvement of the quality of the secretion components of the transplanted SMG and the success rate of keratoplasty, SMG allotransplantation in patients with salivary gland hypofunction, and the reconstruction of the lacrimal gland by tissue engineering are the main directions for future studies on salivary gland transplantation for severe dry eyes.

## Data Availability

All data is included in the manuscript.

## References

[1] Craig J.P., Nichols K.K., Akpek E.K., Caffery B., Dua H.S., Joo C.K., Liu Z., Nelson J.D., Nichols J.J., Tsubota K. (2017). TFOS DEWS II Definition and Classification Report. Ocul. Surf..

[2] Jones L., Downie L.E., Korb D., Benitez-Del-Castillo J.M., Dana R., Deng S.X., Dong P.N., Geerling G., Hida R.Y., Liu Y. (2017). TFOS DEWS II Management and Therapy Report. Ocul. Surf..

[3] Vazirani J., Bhalekar S., Amescua G., Singh S., Basu S. (2020). Minor salivary gland transplantation for severe dry eye disease due to cicatrising conjunctivitis: Multicentre long-term outcomes of a modified technique. Br. J. Ophthalmol..

[4] Filatov V.P., Chevalyev V.E. (1951). Surgical treatment of parenchymatous ophthalmoxerosis. J. Ophthalmol..

[5] Zhang X., Zhu J., Wu Z.M. (1997). Treating xerophthalmia with parotid duct transposition through intraoral incision. West China J. Stomatol..

[6] Agarwal P., Dhakad V., Sharma D. (2019). Feasibility of Parotid Duct Transposition for the Treatment of Dry Eye: A Cadaveric Study. Indian J. Otolaryngol. Head Neck Surg..

[7] Zhang H.C. (1954). Management of length deficiency of parotid duct during transposition. People’s Mil. Surg..

[8] Murube-del-Castillo J. (1986). Transplantation of salivary gland to the lacrimal basin. Scand. J. Rheumatol. Suppl..

[9] Murube J. (2003). Surgical treatment of dry eye. Orbit.

[10] Su J.Z., Zheng B., Liu X.J., Xie Z., Sun D., Cai Z.G., Lv L., Yu G.Y. (2019). Quality of life and patient satisfaction after submandibular gland transplantation in patients with severe dry eye disease. Ocul. Surf..

[11] MacLeod A.M., Robbins S.P. (1992). Submandibular gland transfer in the correction of dry eye. Aust. N. Z. J. Ophthalmol..

[12] Murube J., Marcos M.G., Javate R. (1994). Amylase in mare lacrimale in patients with submandibular salivary gland transplantation to the lacrimal basin. Adv. Exp. Med. Biol..

[13] Geerling G., Sieg P., Bastian G.O., Laqua H. (1998). Transplantation of the autologous submandibular gland for most severe cases of keratoconjunctivitis sicca. Ophthalmology.

[14] Jia G., Wang Y., Lu L., Wang X., Li Z. (1998). Reconstructive lacrimal gland with free submandibular gland transfer for management of xerophthalmia. Zhonghua Yan Ke Za Zhi.

[15] Yu G.Y., Zhu Z.H., Mao C., Cai Z.G., Zou L.H., Lu L., Zhang L., Peng X., Li N., Huang Z. (2004). Microvascular autologous submandibular gland transfer in severe cases of keratoconjunctivitis sicca. Int. J. Oral Maxillofac. Surg..

[16] Paniello R.C. (2007). Submandibular gland transfer for severe xerophthalmia. Laryngoscope.

[17] Borrelli M., Schröder C., Dart J.K.G., Collin J.R.O., Sieg P., Cree I.A., Matheson M.A., Tiffany J.M., Proctor G., van Best J. (2010). Long-term follow-up after submandibular gland transplantation in severe dry eyes secondary to cicatrizing conjunctivitis. Am. J. Ophthalmol..

[18] Zhang L., Su J.Z., Cai Z.G., Lv L., Zou L.H., Liu X.J., Wu J., Zhu Z.H., Mao C., Wang Y. (2019). Factors influencing the long-term results of autologous microvascular submandibular gland transplantation for severe dry eye disease. Int. J. Oral Maxillofac. Surg..

[19] Assy Z., Bikker F.J., Picauly O., Brand H.S. (2022). The association between oral dryness and use of dry-mouth interventions in Sjogren’s syndrome patients. Clin. Oral Investig..

[20] Shiboski C.H., Shiboski S.C., Seror R., Criswell L.A., Labetoulle M., Lietman T.M., Rasmussen A., Scofield H., Vitali C., Bowman S.J. (2017). 2016 American College of Rheumatology/European League Against Rheumatism classification criteria for primary Sjogren’s syndrome: A consensus and data-driven methodology involving three international patient cohorts. Ann. Rheum. Dis..

[21] McGurk M., Combes J. (2013). Controversies in the Management of Salivary Gland Disease.

[22] Geerling G., Collin J.R., Dart J.K. (2009). Ophthalmic experience with submandibular gland transplantation for severe dry eyes. Laryngoscope.

[23] Uchino M., Schaumberg D.A. (2013). Dry Eye Disease: Impact on Quality of Life and Vision. Curr. Ophthalmol. Rep..

[24] Zheng B., Liu X.J., Sun Y.F., Su J.Z., Zhao Y., Xie Z., Yu G.Y. (2017). Development and validation of the Chinese version of dry eye related quality of life scale. Health Qual. Life Outcomes.

[25] (2007). Management and therapy of dry eye disease: Report of the Management and Therapy Subcommittee of the International Dry Eye WorkShop (2007). Ocul. Surf..

[26] Wolffsohn J.S., Arita R., Chalmers R., Djalilian A., Dogru M., Dumbleton K., Gupta P.K., Karpecki P., Lazreg S., Pult H. (2017). TFOS DEWS II Diagnostic Methodology report. Ocul. Surf..

[27] Su J.Z., Cai Z.G., Yu G.Y. (2015). Microvascular autologous submandibular gland transplantation in severe cases of keratoconjunctivitis sicca. Maxillofac. Plast. Reconstr. Surg..

[28] Jacobsen H.C., Hakim S.G., Lauer I., Dendorfer A., Wedel T., Sieg P. (2008). Long-term results of autologous submandibular gland transfer for the surgical treatment of severe keratoconjunctivitis sicca. J. Craniomaxillofac. Surg..

[29] Geerling G., Sieg P. (2008). Transplantation of the major salivary glands. Dev. Ophthalmol..

[30] Wang D.-K., Zhang S.-E., Su Y.-X., Zheng G.-S., Yang W.-F., Liao G.-Q. (2018). Microvascular Submandibular Gland Transplantation for Severe Keratoconjunctivitis Sicca: A Single-Institution Experience of 61 Grafts. J. Oral Maxillofac. Surg..

[31] Su J.-Z., Yu H.-K., Sun Z.-P., Liu X.-J., Cai Z.-G., Lv L., Yu G.-Y. (2017). Effect of computed tomographic venography on donor selection in submandibular gland transplantation in patients with severe dry eye. J. Craniomaxillofac. Surg..

[32] Schroder C., Hakim S.G., Collin J.R., Sieg P., Geerling G. (2003). Long-term follow-up after autologous submandibular gland transplantation in scarring keratoconjunctivitis with absolute dry eyes. Ophthalmologe.

[33] Miyamoto S., Arikawa M., Kagaya Y., Kageyama D., Fukunaga Y. (2020). Large-to-Small End-to-Side Venous Anastomosis in Free Flap Transfer. J. Surg. Res..

[34] Li L., Gao X.L., Song Y.Z., Xu H., Yu G.Y., Zhu Z.H., Liu J.M. (2007). Anatomy of arteries and veins of submandibular glands. Chin. Med. J. (Engl.).

[35] Sant’ Anna A.E., Hazarbassanov R.M., de Freitas D., Gomes J.A. (2012). Minor salivary glands and labial mucous membrane graft in the treatment of severe symblepharon and dry eye in patients with Stevens-Johnson syndrome. Br. J. Ophthalmol..

[36] Luo S.R., Zou L.H., Yan C., Pan Z.Q., Liu J.M., Chen Z.Y., Yin W.H. (2013). Transplantation of autologous labial salivary glands for severe dry eye. Zhonghua Yan Ke Za Zhi.

[37] Su J.Z., Liu X.J., Zhang L., Yu G.Y. (2013). Schirmer test in transplanted submandibular gland: Influencing factors and a modified measurement method. Cornea.

[38] Xu H., Mao C., Liu J.M., Peng X., Zhu Z.H., Yu G.Y. (2011). Microanatomic study of the vascular and duct system of the submandibular gland. J. Oral Maxillofac. Surg..

[39] Ge X.Y., Yu G.Y., Fu J., Wu D.C., Zhang X.X., Wang Y.X., Li S.L. (2012). An experimental study of the management of severe keratoconjunctivitis sicca with autologous reduced-sized submandibular gland transplantation. Br. J. Oral Maxillofac. Surg..

[40] Geerling G., Garrett J.R., Paterson K.L., Sieg P., Collin J.R., Carpenter G.H., Hakim S.G., Lauer I., Proctor G.B. (2008). Innervation and secretory function of transplanted human submandibular salivary glands. Transplantation.

[41] Qin J., Zhang L., Cai Z.G., Mao C., Liu X.J., Lv L., Zou L.H., Peng X., Su J.Z., Wu J. (2013). Microvascular autologous transplantation of partial submandibular gland for severe keratoconjunctivitis sicca. Br. J. Ophthalmol..

[42] Su J.Z., Yang N.Y., Liu X.J., Cai Z.G., Lv L., Zhang L., Wu L.L., Liu D.G., Ren W.G., Gao Y. (2014). Obstructive sialadenitis of a transplanted submandibular gland: Chronic inflammation secondary to ductal obstruction. Br. J. Ophthalmol..

[43] Shi L., Cong X., Zhang Y., Ding C., Ding Q.W., Fu F.Y., Wu L.L., Yu G.Y. (2010). Carbachol improves secretion in the early phase after rabbit submandibular gland transplantation. Oral Dis..

[44] Yang N.Y., Ding C., Li J., Zhang Y., Xiang R.L., Wu L.L., Yu G.Y., Cong X. (2017). Muscarinic acetylcholine receptor-mediated tight junction opening is involved in epiphora in late phase of submandibular gland transplantation. J. Mol. Histol..

[45] Ding C., Cong X., Zhang Y., Yang N.Y., Li S.L., Wu L.L., Yu G.Y. (2014). Hypersensitive mAChRs are involved in the epiphora of transplanted glands. J. Dent. Res..

[46] Shan X.F., Lv L., Cai Z.G., Yu G.Y. (2019). Botulinum toxin A treatment of epiphora secondary to autologous submandibular gland transplantation. Int. J. Oral Maxillofac. Surg..

[47] Cong X., Zhang Y., Li J., Mei M., Ding C., Xiang R.L., Zhang L.W., Wang Y., Wu L.L., Yu G.Y. (2015). Claudin-4 is required for modulation of paracellular permeability by muscarinic acetylcholine receptor in epithelial cells. J. Cell Sci..

[48] Ding C., Cong X., Zhang X.M., Li S.L., Wu L.L., Yu G.Y. (2017). Decreased interaction between ZO-1 and occludin is involved in alteration of tight junctions in transplanted epiphora submandibular glands. J. Mol. Histol..

[49] Zhang Y., Xiang B., Li Y.M., Wang Y., Wang X., Wang Y.N., Wu L.L., Yu G.Y. (2006). Expression and characteristics of vanilloid receptor 1 in the rabbit submandibular gland. Biochem. Biophys. Res. Commun..

[50] Zhang Y., Cong X., Shi L., Xiang B., Li Y.M., Ding Q.W., Ding C., Wu L.L., Yu G.Y. (2010). Activation of transient receptor potential vanilloid subtype 1 increases secretion of the hypofunctional, transplanted submandibular gland. Am. J. Physiol. Gastrointest. Liver Physiol..

[51] Cong X., Zhang Y., Yang N.Y., Li J., Ding C., Ding Q.W., Su Y.C., Mei M., Guo X.H., Wu L.L. (2013). Occludin is required for TRPV1-modulated paracellular permeability in the submandibular gland. J. Cell Sci..

[52] Cong X., Zhang Y., Shi L., Yang N.Y., Ding C., Li J., Ding Q.W., Su Y.C., Xiang R.L., Wu L.L. (2012). Activation of transient receptor potential vanilloid subtype 1 increases expression and permeability of tight junction in normal and hyposecretory submandibular gland. Lab. Investig..

[53] Su J.Z., Liu X.J., Wang Y., Cai Z.G., Zhang L., Lv L., Wang Z., Hong X., Yu G.Y. (2016). Effects of Capsaicin and Carbachol on Secretion From Transplanted Submandibular Glands and Prevention of Duct Obstruction. Cornea.

[54] Geerling G., Honnicke K., Schroder C., Framme C., Sieg P., Lauer I., Pagel H., Kirschstein M., Seyfarth M., Marx A.M. (2000). Quality of salivary tears following autologous submandibular gland transplantation for severe dry eye. Graefes Arch. Clin. Exp. Ophthalmol..

[55] Cai J.R., Shan X.F., Cai Z.G., Zhang X., Yu G.Y. (2014). A new treatment for epiphora secondary to submandibular gland transplantation: Transcutaneous atropine gel. Ocul. Surf..

[56] Zhang L., Zhu Z.H., Dai H.J., Cai Z.G., Mao C., Peng X., Yu G.Y. (2007). Application of 99mTc-pertechnetate scintigraphy to microvascular autologous transplantation of the submandibular gland in patients with severe keratoconjunctivitis sicca. J. Nucl. Med..

[57] Su J.Z., Cai Z.G., Liu X.J., Lv L., Yu G.Y. (2018). Management of duct obstruction in transplanted submandibular glands. J. Craniomaxillofac. Surg..

[58] Geerling G., Daniels J.T., Dart J.K., Cree I.A., Khaw P.T. (2001). Toxicity of natural tear substitutes in a fully defined culture model of human corneal epithelial cells. Investig. Ophthalmol. Vis. Sci..

[59] Ge X.Y., Yu G.Y., Cai Z.G., Mao C. (2006). Establishment of submandibular gland allotransplantation model in miniature swine. Chin. Med. J. (Engl.).

[60] Almansoori A.A., Khentii N., Kim B., Kim S.M., Lee J.H. (2019). Mesenchymal Stem Cell Therapy in Submandibular Salivary Gland Allotransplantation: Experimental Study. Transplantation.

[61] Almansoori A.A., Khentii N., Ju K.W., Kim B., Kim S.M., Lee J.H. (2020). FK506 immunosuppression for submandibular salivary gland allotransplantation in rabbit. J. Korean Assoc. Oral Maxillofac. Surg..

[62] Jacobsen H.C., Hakim S.G., Trenkle T., Nitschke M., Steven P., Sieg P. (2013). Allogenic submandibular gland transplantation following hematopoietic stem cell transplantation. J. Craniomaxillofac. Surg..

[63] Dietrich J., Massie I., Roth M., Geerling G., Mertsch S., Schrader S. (2016). Development of Causative Treatment Strategies for Lacrimal Gland Insufficiency by Tissue Engineering and Cell Therapy. Part 1: Regeneration of Lacrimal Gland Tissue: Can We Stimulate Lacrimal Gland Renewal In Vivo?. Curr. Eye Res..

[64] Hirayama M., Tsubota K., Tsuji T. (2017). Generation of a Bioengineered Lacrimal Gland by Using the Organ Germ Method. Methods Mol. Biol..

[65] Hirayama M., Ogawa M., Oshima M., Sekine Y., Ishida K., Yamashita K., Ikeda K., Shimmura S., Kawakita T., Tsubota K. (2013). Functional lacrimal gland regeneration by transplantation of a bioengineered organ germ. Nat. Commun..

[66] Lin H., Sun G., He H., Botsford B., Li M., Elisseeff J.H., Yiu S.C. (2016). Three-Dimensional Culture of Functional Adult Rabbit Lacrimal Gland Epithelial Cells on Decellularized Scaffold. Tissue Eng. Part A.

[67] Hirayama M., Kawakita T., Tsubota K., Shimmura S. (2016). Challenges and Strategies for Regenerating the Lacrimal Gland. Ocul. Surf..

[68] Hirayama M. (2018). Advances in Functional Restoration of the Lacrimal Glands. Investig. Ophthalmol. Vis. Sci..

[69] Massie I., Spaniol K., Barbian A., Geerling G., Metzger M., Schrader S. (2018). Development of lacrimal gland spheroids for lacrimal gland tissue regeneration. J. Tissue Eng. Regen. Med..

[70] Ackermann P., Hetz S., Dieckow J., Schicht M., Richter A., Kruse C., Schroeder I.S., Jung M., Paulsen F.P. (2015). Isolation and Investigation of Presumptive Murine Lacrimal Gland Stem Cells. Investig. Ophthalmol. Vis. Sci..

[71] Xie C., Li X.Y., Cui H.G. (2015). Potential candidate cells for constructing tissue-engineered lacrimal duct epithelium: A histological and cytological study in rabbits. J. Zhejiang Univ. Sci. B.

[72] Massie I., Spaniol K., Barbian A., Poschmann G., Stuhler K., Geerling G., Metzger M., Mertsch S., Schrader S. (2017). Evaluation of Decellularized Porcine Jejunum as a Matrix for Lacrimal Gland Reconstruction In Vitro for Treatment of Dry Eye Syndrome. Investig. Ophthalmol. Vis. Sci..

